# The Clinical, Myopathological, and Genetic Analysis of 20 Patients With Non-dystrophic Myotonia

**DOI:** 10.3389/fneur.2022.830707

**Published:** 2022-03-08

**Authors:** Quanquan Wang, Zhe Zhao, Hongrui Shen, Qi Bing, Nan Li, Jing Hu

**Affiliations:** ^1^Department of Neuromuscular Disorders, The Third Hospital of Hebei Medical University, Shijiazhuang, China; ^2^Department of Neurology, Qilu Hospital of Shandong University, Qingdao, China

**Keywords:** non-dystrophic myotonia, myotonia congenita, paramyotonia congenita, *CLCN1*, *SCN4A*

## Abstract

**Introduction:**

Non-dystrophic myotonias (NDMs) are skeletal muscle ion channelopathies caused by *CLCN1* or *SCN4A* mutations. This study aimed to describe the clinical, myopathological, and genetic analysis of NDM in a large Chinese cohort.

**Methods:**

We reviewed the clinical manifestations, laboratory results, electrocardiogram, electromyography, muscle biopsy, genetic analysis, treatment, and follow-up of 20 patients (from 18 families) with NDM.

**Results:**

Cases included myotonia congenita (MC, 17/20) and paramyotonia congenita (PMC, 3/20). Muscle stiffness and hypertrophy, grip and percussion myotonia, and the warm-up phenomenon were frequently observed in MC and PMC patients. Facial stiffness, eye closure myotonia, and cold sensitivity were more common in PMC patients and could be accompanied by permanent weakness. Nine MC patients and two PMC patients had cardiac abnormalities, mainly manifested as cardiac arrhythmia, and the father of one patient died of sudden cardiac arrest. Myotonic runs in electromyography were found in all patients, and seven MC patients had mild myopathic changes. There was no difference in muscle pathology between MC and PMC patients, most of whom had abnormal muscle fiber type distribution or selective muscle fiber atrophy. Nineteen *CLCN1* variants were found in 17 MC patients, among which c.795T>G (p.D265E) was a new variant, and two *SCN4A* variants were found in three PMC patients. The patients were treated with mexiletine and/or carbamazepine, and the symptoms of myotonia were partially improved.

**Conclusions:**

MC and PMC have considerable phenotypic overlap. Genetic investigation contributes to identifying the subtype of NDM. The muscle pathology of NDM lacks specific changes.

## Introduction

Non-dystrophic myotonias (NDMs) are a group of skeletal muscle disorders that have myotonia as their common feature, in reference to a delayed muscle relaxation after voluntary or evoked muscle contraction (1). NDMs present clinically as muscle stiffness and muscle hypertrophy (a body-builder appearance), which may be accompanied by muscle weakness (paroxysmal or permanent) and myalgia. The onset of symptoms usually occurs in the first or second decade of life. The phenotype of NDMs almost exclusively involves the skeletal muscle, and no relevant extra-muscular involvement has been clearly described to date. NDMs are different from dystrophic myotonias (DMs) because of the absence of muscle atrophy and systemic features (2–4). According to the characteristics of clinical manifestations, NDMs are divided into myotonia congenita (MC), paramyotonia congenita (PMC), and sodium channel myotonia (SCM). The typical clinical features of patients with MC include muscle stiffness, grip and percussion myotonia, and the warm-up phenomenon (myotonia relieved after repeated activity). Patients with PMC show cold sensitivity, exercise-induced myotonia, myotonia worsening after repetitive activity, episodic weakness, and onset before the age of 10. SCM is clinically rare and has some “atypical” clinical manifestations that are lack of cold sensitivity and episodic weakness, including potassium-aggravated myotonia, fluctuating myotonia, permanent myotonia, and acetazolamide-induced myotonia (2, 5–7). However, the clinical symptoms are sometimes atypical, which makes it difficult to distinguish the clinical phenotype of NDMs.

MC caused by *CLCN1* mutations that encode the voltage-gated chloride channel (ClC-1) in the skeletal muscle is classified into the autosomal dominant (AD) Thomsen's myotonia congenita (DMC) and the autosomal recessive (AR) Becker's myotonia congenita (RMC) according to the mode of inheritance. The *CLCN1* gene located at chromosome 7q35 contains 23 exons and encodes 988 amino acids (8). PMC and SCM are allelic and AD disorders caused by *SCN4A* mutations which encode the α-subunit of the voltage-gated sodium channel (Na_V_1.4) in the skeletal muscle. The *SCN4A* gene located at chromosome 17q23.1-25.3 contains 24 exons and encodes 1,836 amino acids (9). More than 200 mutations in *CLCN1* and 67 mutations in *SCN4A* have been linked to NDMs (10, 11). *CLCN1* or *SCN4A* mutations lead to the dysfunction of the CLC-1 or Na_V_1.4 channel, which may predispose the muscle to sarcolemmal hyperexcitability manifesting as myotonia (4).

Due to the rarity of NDMs, large Chinese cohorts with NDMs have not been described in the literature to date, and most reports have been on single to several patients or families (6, 12, 13). Here, we reviewed the clinical manifestations, laboratory results, electrocardiogram (ECG), electromyography (EMG), muscle biopsy, genetic analysis, treatment, and follow-up of 20 patients with NDMs (from 18 families), aiming to explore the clinical, myopathological, and genetic characteristics of NDMs in Chinese people.

## Methods

### Patients

We retrospectively reviewed the clinical, laboratory, ECG, EMG, muscle biopsy, genetic features, treatment, and follow-up of 20 patients (from 18 families) with NDMs, whose main clinical manifestation was myotonia, with genetic testing revealing *CLCN1* or *SCN4A* mutations, at the Third Hospital of Hebei Medical University in China between June 2010 and December 2020. The parents of all of the patients were anamnetically and/or clinically examined, and genetic testing was performed on all of the available relatives of the patients for family verification. Written informed consent was obtained from the patients or their legal relatives before skeletal muscle biopsy and genetic analysis. This study was approved by the ethics committee of the Third Hospital of Hebei Medical University and was performed in accordance with the Declaration of Helsinki.

### Electrophysiological Study

Needle EMG and nerve conduction studies were performed on all 20 patients.

### Muscle Biopsy and Histochemistry Stains

Open muscle biopsy was performed on the bicep brachii muscles under local infiltration anesthesia. The specimens were flash-frozen in isopentane chilled by liquid nitrogen. Serial frozen sections (7 μm) were stained with hematoxylin and eosin (HE), modified Gomori trichrome (MGT), nicotinamide adenine dinucleotide-tetrazolium reductase (NADH-TR), succinic dehydrogenase (SDH), adenosine monophosphate deaminase (AMP), cytochrome c oxidase (CCO), acid phosphatase (ACID), adenosine triphosphatase (ATPase, pH 4.5, 4.7, and 10.1), periodic acid–Schiff (PAS), oil red O (ORO), and Sudan black B (SBB). The stained tissues were observed under a light microscope for histopathological analysis.

### Genetic Testing

Genomic DNA (gDNA) was extracted from peripheral blood (collected from all of the patients and some of their family members) with a DNA kit, referring to the kit instructions for specific steps. All of the exons and exon–intron boundaries (at least 50 intronic nucleotides) of 12 ion channelopathy genes (*CACNA1A, CACNA1S, CLCN1, KCNA1, KCNE3, KCNH2, KCNJ2, KCNJ5, KCNJ16, KCNJ18, KCNQ1*, and *SCN4A)* were sequenced by next-generation sequencing (NGS) (My Genostics, Beijing, China). The sequencing data were aligned to human reference genome 19 with the BWA program. Single-nucleotide variants (SNVs) and small insertions/deletions (indels) were detected and genotyped using GATK software. Annotations of the variants were performed with the HGMD (http://www.hgmd.cf.ac.uk/ac/index.php), OMIM (https://www.omim.org/), and ClinVar (https://www.ncbi.nlm.nih.gov/clinvar/) database. The SNVs/indels were filtered if they showed >5% frequency in several databases, including dbSNP (https://www.ncbi.nlm.nih.gov/snp), 1,000 Genomes (https://www.internationalgenome.org/), ExAC (http://exac.broadinstitute.org/), and the 1,000 in-house Asia database (i.e., generated by NGS of gDNA from 1,000 normal Chinese individuals, and provided by MyGenostics, Inc.). The probable pathogenic variants were then predicted using SIFT (http://sift.jcvi.org/), PolyPhen-2 (http://genetics.bwh.harvard.edu/pph2/), and MutationTaster (http://www.mutationtaster.org/). Sanger sequencing was used to verify the variants identified by NGS and to examine the available relatives of the patients. The American College of Medical Genetics and Genomics (ACMG) standards were used to evaluate the pathogenicity (14).

### Treatment and Follow-Up

The patient's specific medication, efficacy, and 1-year follow-up (telephone or outpatient) period data were collected. The efficacy of antimyotonic treatment was evaluated according to the level of the patient's perceived efficacy (the impact of myotonia on daily life activities) and clinical assessment (grip and percussion myotonia, myotonia relieved after repeated activity), documented by the treating physician.

## Results

### Clinical Features

The detailed clinical, laboratory, ECG, and EMG data of the 20 patients with NDMs are shown in Table 1.

**Table 1:** Clinical, laboratory, electrocardiogram, and electromyography data of 20 patients with non-dystrophic myotonia.

**Pt. ID**	**Onset/first-diagnosis/cardiac involvement age (y)**	**Sex/family history**	**Skeletal muscle**												**Cardiac**		**Phenotype**
			**Myotonia**		**Muscle hypertrophy**	**Myalgia**	**Cold sensitivity/ warm-up**	**Percussion myotonia**	**Grip myotonia**	**Muscle force**	**Joint contracture**	**CK (IU/L)**	**EMG**		**Symptoms**	**ECG/ Holter**	
			**Limbs**	**Face**									**Myotonic runs**	**Myopathic changes**			
1	9/15/15	M/+	+	–	+	–	+/+	+	+	5	–	227	+++	–	The father died of sudden cardiac arrest	Sinus tachycardia	DMC
2	19/21/21	M/+	+	–	–	+	+/+	+	+	5	–	–	+++	–	Palpitations, chest tightness	Premature atrial beats	DMC
3	3/4/4	M/–	+	–	+	–	+/+	+	+	5	+	423	+++	+	–	Sinus tachycardia	DMC
4	53.5/54/54	F/–	+	–	–	–	+/+	–	+	5	–	276	++	–	–	Premature ventricular beats	DMC
5	9/12/18	M/+	+	+	+	–	–/+	+	+	5	+	142	+++	+	Palpitations	Premature atrial beats	DMC
6	9/10/–	M/+	+	–	+	–	–/+	+	+	5	–	–	+++	+	–	–	DMC
7	1/5/–	M/–	+	–	+	–	–/+	+	+	5	+	–	+++	+	–	–	DMC
8	15/20/–	M/+	+	–	+	–	–/+	–	+	5	–	–	+++	–	–	–	DMC
9	10/14/–	M/+	+	–	+	–	–/+	+	+	5	–	484	++	–	–	–	DMC
10	27/27/–	M/–	+	–	–	+	+/+	+	–	5	–	–	++	–	–	–	DMC
11	12/14/18	F/–	+	+	–	+	+/+	–	+	5	–	189	++	–	Palpitations, chest pain	Premature atrial beats, premature ventricular beats	RMC
12	9/12/12	M/–	+	–	+	–	+/+	+	+	5	+	–	+++	+	–	Cardiac arrhythmia	RMC
13	2/4/4	F/–	+	–	+	–	+/+	+	+	5	–	–	+++	+	–	Cardiac arrhythmia	RMC
14	1/14/18	M/–	+	–	+	–	+/+	+	+	5	–	350	+++	–	Palpitations	Elevated heart rhythm variability	RMC
15	5/8/–	M/+	+	–	+	–	–/+	+	+	5	–	–	+++	+	–	–	RMC
16	7/13/–	F/+	+	–	+	–	–/+	+	+	5	+	–	+++	–	–	–	RMC
17	5/20/–	M/–	+	–	+	–	–/+	+	+	5	–	172	+++	–	–	–	RMC
18	2/4/13	F/–	+	+	+	–	+/+	+	+	5	+	821	+++	–	Chest tightness	Premature atrial beats, sinus tachycardia	PMC
19	5/20/20	M/–	+	+	+	–	+/+	+	+	4	+	1,620.4	+++	–	Chest tightness, chest pain	Complete right bundle branch block	PMC
20	1/5/–	M/–	+	+	+	–	+/+	+	+	5	+	–	+++	–	–	–	PMC

*–, Negative or normal; +, positive; /, unchecked. K^+^, potassium (reference interval, 3.5–5.5 mmol/L); CK, creatine kinase [reference interval, 25–130 U/L or 50–310 IU/L (Pt. ID: 10, 12, 18, 19, 20)]; ECG, electrocardiogram; EMG, electromyography; Holter, 24-h Holter; y, year(s); M, male; F, female; DMC, autosomal dominant Thomsen's myotonia congenita; RMC, autosomal recessive Becker's myotonia congenita; PMC, paramyotonia congenita. Patients 5 and 6 were from one family; patients 15 and 16 were from one family*.

**Figure 1:**
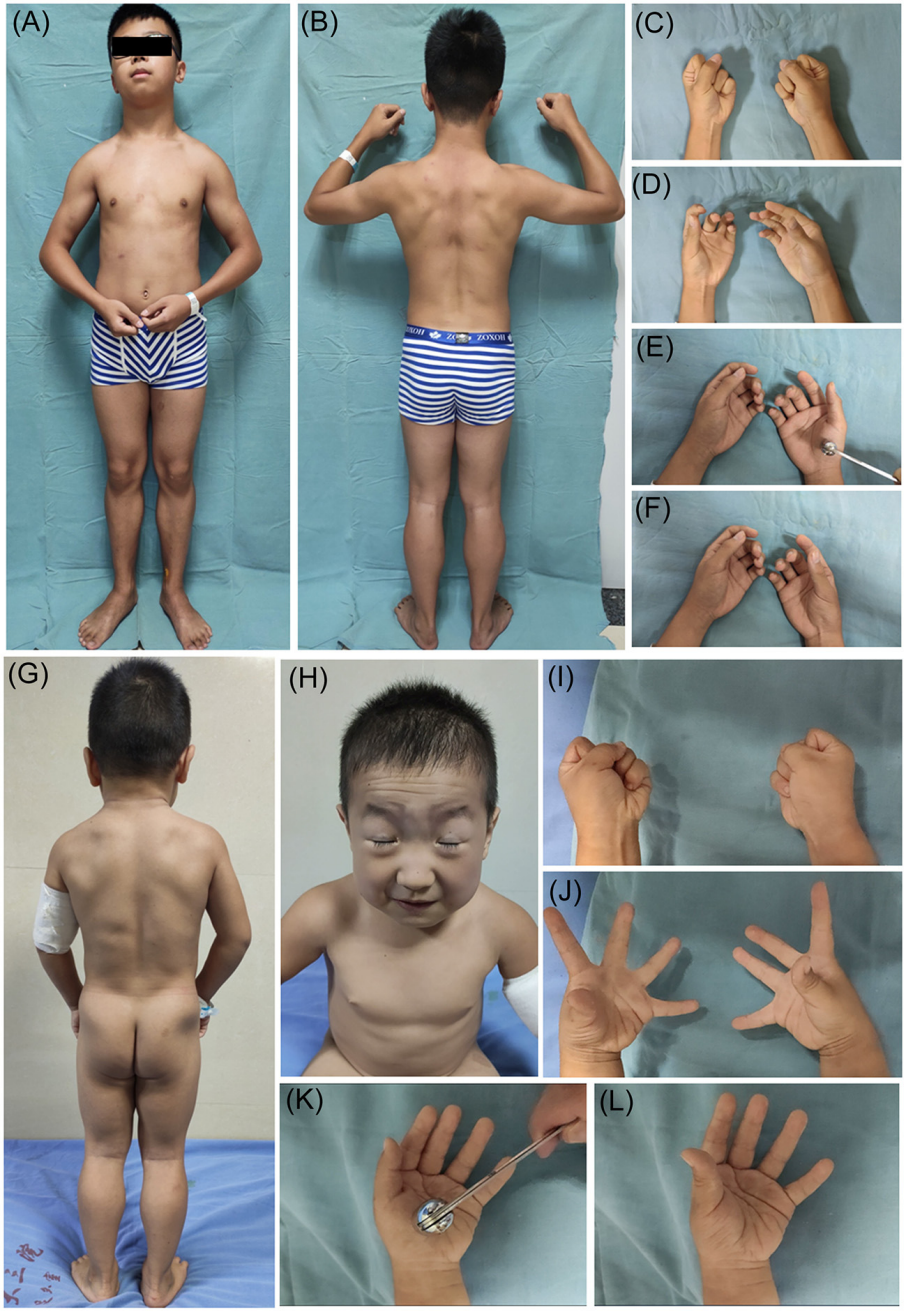
Clinical features of non-dystrophic myotonia. Patient 17 (myotonia congenita): **(A,B)** show muscle stiffness and hypertrophy; **(C,D)** show grip myotonia; **(E,F)** show percussion myotonia. Patient 20 (paramyotonia congenita); **(G)** shows muscle stiffness and hypertrophy; **(H)** shows facial stiffness and eye closure myotonia; **(I,J)** show grip myotonia; **(K,L)** show percussion myotonia.

This study included 17 patients with MC (DMC 10/17, RMC 7/17) and three patients with PMC. Fifteen patients were male, the other five patients were female, and eight of them had a positive family history of myotonia. The mean age at onset of MC was 11.6 ± 12.3 years, ranging from 1 to 53.5 years, and the mean age at onset of PMC was 2.7 ± 1.7 years, ranging from 1 to 5 years. Muscle stiffness and hypertrophy, grip and percussion myotonia, and the warm-up phenomenon, as well as secondary joint contracture, were frequently observed in MC and PMC patients. Facial stiffness and eye closure myotonia were observed in all patients with PMC and could be accompanied by myotonia in the tongue, throat, and neck muscles, while facial stiffness was observed in two patients with MC. Cold worsened myotonia in all patients with PMC and nine patients with MC. Three patients had MC accompanied by myalgia, and one patient had PMC accompanied by permanent weakness. The clinical features of MC and PMC patients are shown in Figure 1.

Nine patients with MC and two patients with PMC had cardiac abnormalities, mainly manifested as cardiac arrhythmia on the ECG or 24-h Holter including two with premature atrial beats, one with premature ventricular beats, two with sinus tachycardia, two with premature atrial beats and premature ventricular beats or sinus tachycardia, two with sinus arrhythmia, one with complete right bundle branch block, and one with elevated heart rhythm variability. Among them, six patients complained of palpitations, chest tightness, chest pain, and related symptoms, and the father of one patient died from sudden cardiac arrest. The average age of cardiac involvement was 17.9 ± 12.7 years, which appeared either before or after the onset of muscle stiffness. ECG demonstrated neither underlying cardiomyopathy nor structural heart disease in any patient. Four patients were treated with antimyotonic medications without other medications when ECG or Holter examination was performed. The detailed cardiologic data of 11 patients with NDM are shown in Supplementary Table 1.

Creatine kinase (CK) was normal or slightly elevated. Myotonic runs in EMG were found in all of the patients, and seven patients with MC had mild myopathic changes, while PMC patients did not have such changes.

### Muscle Pathological Analysis

**Figure 2:**
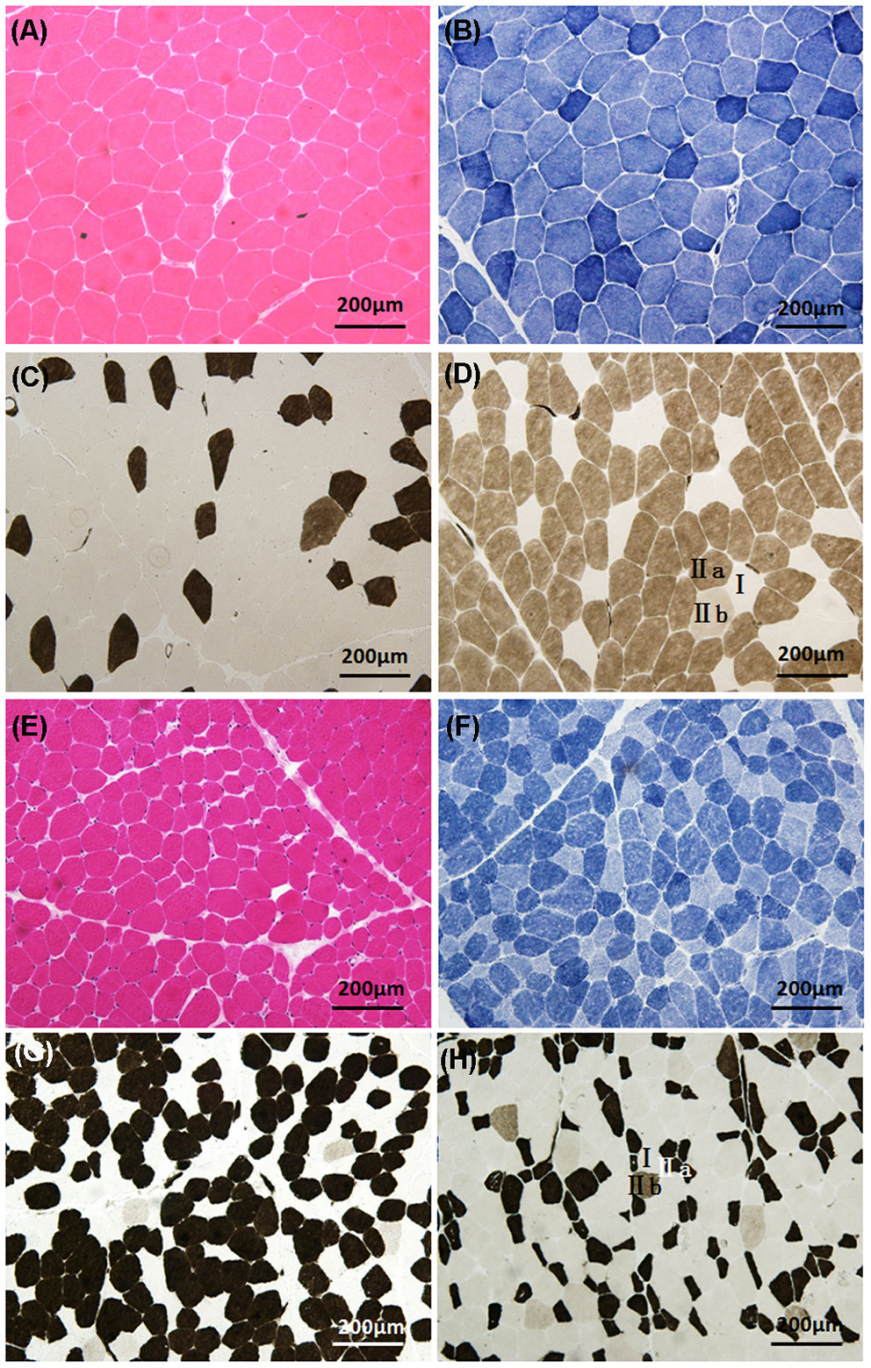
Histologic characteristics of non-dystrophic myotonias. Patient 5 (myotonia congenita): **(A)** (HE) demonstrates the variation of fiber diameter; **(B)** (NADH-TR) shows that the enzyme activity was normal; **(C,D)** (ATPase, pH 4.5 and 10.1) demonstrate the predominance of type IIa fibers, atrophy of type I fibers, and reduction of type IIb fibers. Patient 20 (paramyotonia congenita): **(E)** (HE) demonstrates the variation of fiber diameter; **(F)** (NADH-TR) shows that the enzyme activities were focally decreased in some fibers; **(G,H)** (ATPase, pH 4.5 and 10.1) demonstrate the predominance of type I fibers, atrophy of type IIa fibers, and reduction of type IIb fibers.

Pathological analysis of skeletal muscle biopsy was performed on 17 patients with NDMs (Figure 2). Fourteen patients showed variation of fiber diameter, normal or mildly proliferative connective tissue in HE and MGT. Among them, five patients showed occasional degeneration or necrosis, two showed an increase in the number of internal nuclei, and two had opaque muscle fibers. The enzyme activities of NADH-TR, SDH, and CCO were focally decreased in some fibers of five patients. Myosin ATPase (pH = 4.5, 4.7, 10.1) staining showed abnormal muscle fiber type distribution or selective muscle fiber atrophy in 14 patients. Among them, eight patients demonstrated predominance of type II fibers (predominance of type IIa fibers and absence or reduction of type IIb fibers) with or without atrophy of type I fibers. Two patients showed predominance of type I fibers, with one case presenting atrophy of type IIa fibers and reduction of type IIb fibers. Two patients only showed atrophy of type I fibers, and one patient showed only atrophy of type II fibers. One patient displayed two-type fiber grouping. The muscle pathology of the other three patients was almost normal, and no special structure was found. The detailed muscle biopsy data of 17 patients with NDM are shown in Supplementary Table 2.

### Genetic Analysis

Sequencing showed that 19 different pathogenic variants in the *CLCN1* gene were identified in 17 MC patients, including 14 missense, four frameshift, and one non-sense variants. Among them, c.795T>G (p.D265E) was a novel missense variant, and the other 18 variants had previously been reported (6, 12, 15–17). Combining this with family history, 10 patients with one heterozygous variant and seven patients with two compound heterozygous variants were diagnosed with DMC and RMC, respectively. Two heterozygous missense variants in the *SCN4A* gene, which had been reported in the literature, were identified in three PMC patients (7, 18, 19). The pathogenicity of gene variants was judged by the ACMG standards, and all of them were pathogenic or likely pathogenic (for arguments in favor of pathogenicity for all their variants and the location on the CLC-1 or Na_V_1.4 protein for the missense variants, see Supplementary Table 3) (14). Genetic results are listed in Table 2.

**Table 2:** The gene mutation information of 20 patients with non-dystrophic myotonia.

**Pt. ID**	**Phenotype**	**Gene**	**Coding channel**	**Exon**	**Nucleotide change**	**Protein change**	**Mutation type**	**Variant classification (ACMG)**	**Variant source**
1	DMC	*CLCN1*	ClC-1	8	c.T920C	p.F307S	Missense	P	Father
2	DMC	*CLCN1*	ClC-1	22	c.2527C>T	p.L843F	Missense	LP	Mother
3	DMC	*CLCN1*	ClC-1	8	c.892G>A	p.A298T	Missense	P	Spontaneous
4	DMC	*CLCN1*	ClC-1	3	c.350A>G	p.D117G	Missense	P	Spontaneous
5	DMC	*CLCN1*	ClC-1	12	c.1261dupC	p.R421fs	Frameshift	P	Mother
6	DMC	*CLCN1*	ClC-1	12	c.1261dupC	p.R421fs	Frameshift	P	Mother
7	DMC	*CLCN1*	ClC-1	15	c.1679T>C	p.M560T	Missense	P	Spontaneous
8	DMC	*CLCN1*	ClC-1	2	c.214_215delAG	p.R72fs	Frameshift	P	Mother
9	DMC	*CLCN1*	ClC-1	19	c.2362C>T	p.Q788X	Missense	LP	Mother
10	DMC	*CLCN1*	ClC-1	3	c.350A>G	p.D117G	Missense	P	Spontaneous
11	RMC	*CLCN1*	ClC-1	8.18	c.892G>A	p.A298T	Missense	P	Father
					c.2207C>T	p. T736I	Missense	LP	Mother
12	RMC	*CLCN1*	ClC-1	3.12	c.433G>T	p.A145S	Missense	P	Father
					c.1277C>A	p.T426N	Missense		Mother
13	RMC	*CLCN1*	ClC-1	6.8	c.762C>G	p.C254W	Missense	LP	Mother
					c.962T>A	p.V321E	Missense		Father
14	RMC	*CLCN1*	ClC-1	7.16	**c.795T>G**	**p.D265E**	Missense	LP	Father
					c.1872G>T	p.E624D	Missense		Mother
15	RMC	*CLCN1*	ClC-1	8.9	c.857T>A	p.V286E	Missense	LP	Father
					c.1012C>T	p.R338^*^	Nonsense	P	Mother
16	RMC	*CLCN1*	ClC-1	8.9	c.857T>A	p.V286E	Missense	LP	Father
					c.1012C>T	p.R338^*^	Nonsense	P	Mother
17	RMC	*CLCN1*	ClC-1	12.19	c.1389ins T	p.F463fs	Frameshift	P	Father
					c.2330del G	p.G777fs	Frameshift		Mother
18	PMC	*SCN4A*	Na_V_1.4	22	c.3877G>A	p.V1293I	Missense	P	Spontaneous
19	PMC	*SCN4A*	Na_V_1.4	13	c.2065C>T	p.L689F	Missense	P	Spontaneous
20	PMC	*SCN4A*	Na_V_1.4	13	c.2065C>T	p.L689F	Missense	P	Spontaneous

*Bold text, novel mutation*.

*, *a premature translational stop signal. ACMG, The American College of Medical Genetics and Genomics; P, pathogenic; LP, likely pathogenic; DMC, autosomal dominant Thomsen's myotonia congenita; RMC, autosomal recessive Becker's myotonia congenita; PMC, paramyotonia congenita. Patients 5 and 6 were from one family; patients 15 and 16 were from one family*.

### Treatment and Follow-Up

All of the patients were treated with mexiletine (50–100 mg, 3 × /day) and/or carbamazepine (100–200 mg, 2–3 × /day) orally. After treatment, their limbs were more flexible than before, and myotonia symptoms were partially improved. The patients' symptoms were stable during the 1-year follow-up period. None of the treated patients developed intolerable or severe side effects requiring drug discontinuation or dose reduction.

## Discussion

NDMs are a group of hereditary skeletal muscle ion channelopathies characterized by myotonia and caused by *CLCN1* or *SCN4A* variants, including MC, PMC, and SCM. This study examined 20 patients with NDMs, whose main clinical manifestations were muscle stiffness without muscle atrophy, confirmed by genetic testing, including 17 patients with MC and three patients with PMC.

### Clinical Phenotypes

Myotonia is a common clinical manifestation of the skeletal muscle ion channelopathy, which is usually classified into DM and NDM (MC, PMC, SCM). NDM is usually accompanied by muscle stiffness and hypertrophy and typical myotonia discharge at rest with no or mild myopathic potentials in EMG, which is different from DM.

In our study, the incidence of PMC (3/20, 15%) was significantly lower than that of MC (17/20, 85%), which was consistent with previous reports (20). MC is classified into the AD-Thomsen type (DMC) and the AR-Becker type (RMC). The incidence of RMC is higher than that of DMC in Western nations (21, 22), while the incidence of DMC (10/17, 59%) was higher than that of RMC (7/17, 41%) in our study, in agreement with several studies in Asia (20, 23), which might be due to racial differences. The age at onset of NDM is usually <20 years. In this study, only two patients with DMC caused by *CLCN1* c.350A>G variant had an older age of onset, 27 and 53.5 years, respectively, which might be related to the *CLCN1* variant site. PMC had an earlier age of onset at 2.7 ± 1.7 years compared to 11.6 ± 12.3 years for MC, which is in agreement with previous reports (24). Muscle stiffness was the most prominent symptom of NDMs, and facial stiffness and eye closure myotonia were more common in PMC than in MC (4, 24). MC patients are usually characterized by the warm-up phenomenon, while PMC patients typically show worsening of their myotonia after repetitive movements and in low-temperature environments. However, in our study, PMC patients also had a warm-up phenomenon similar to MC patients, and similar observations were reported by some previous studies (4), which suggests that this feature needs to be revised as a clinical standard for NDM classification. In addition, cold sensitivity is more common in PMC, but it is not unique to PMC and can also be seen in MC (4). MC and PMC have considerable phenotypic overlap. In addition, PMC may be accompanied by episodic or permanent weakness. In our study, PMC with the L689F variant was accompanied by progressive muscle weakness, which was characterized by difficulty in climbing stairs, and similar muscle weakness has been reported for the same variant (7). In eight patients, myotonia could cause secondary dystonia, which could lead to secondary joint contractures during the development of the disease (15).

Nine patients with MC and two patients with PMC had cardiac abnormalities, mainly manifested as cardiac arrhythmia. Among them, six patients complained of palpitations, chest tightness, chest pain, and related symptoms, and the father of one patient died from sudden cardiac death. The average age of heart involvement was 17.9 ± 12.7 years, which could occur either before or after the onset of muscle stiffness. It was originally reported that the expression of the *SCN4A* and *CLCN1* genes was limited to the skeletal muscle (25), but subsequent studies have shown that they are also slightly expressed in the normal human heart (26–28). A study reported that *SCN4A* variants caused cardiac arrhythmias and Brugada syndrome (29), and a German study found that six MC patients with *CLCN1* variants had cardiac arrhythmias or conduction defects, and three of them had pacemaker implanted (4), but the type and severity of cardiac abnormalities observed in the different studies (including the present study) were very heterogeneous. The causal relation between the *CLCN1* and *SCN4A* variants and cardiac manifestations remains debated or possibly coincidental. However, NDM patients must undergo regular cardiac evaluation, as the intake of anti-myotonic drugs can unmask latent and potentially life-threatening arrhythmias.

The serum CK level of NDM patients was normal to slightly elevated. Among them, 10 patients had slightly elevated levels, which may have been due to long-standing myotonia or excessive muscle contraction. Myotonic runs in EMG were found in all patients, and seven MC patients had mild myopathic potentials. A comparison of clinical features between MC and PMC is shown in Table 3.

**Table 3:** Comparison of clinical features between myotonia congenita and paramyotonia congenita.

		**MC(*n* = 17)**	**PMC(*n* = 3)**
Gender (M:F)		13:4	2:1
Family history (+)		8/17	0/3
Age of onset (y)		11.6 ± 12.3	2.7 ± 1.7
Myotonia	Limbs	17	3
	Face	2	3
Muscle hypertrophy		13	3
Myalgia		3	0
Percussion myotonia		14	3
Grip myotonia		16	3
Cold sensitivity		9	3
Warm-up		17	3
Permanent weakness		0	1
Joint contracture		5	3
Cardiac involvement		9	2
EMG	Myotonic runs	17	3
	Myopathic potentials	7	0

*MC, myotonia congenita; DMC, autosomal dominant Thomsen's myotonia congenita; RMC, autosomal recessive Becker's myotonia congenita; PMC, paramyotonia congenita; M, male; F, female; y, year(s); EMG, electromyography*.

### Muscle Pathology

The muscle pathologies of NDMs are usually normal or limited to mild myopathic changes (7, 30). Variation of fiber size and type II predominancy with a reduction or absence of type IIB fibers were commonly observed in several cases with NDMs. Other findings, including internal nuclei, muscle fiber atrophy, and myopathy-like pathological changes, have been reported (7, 31–33). This study found that many patients showed variation of fiber diameter, abnormal muscle fiber type distribution, or selective muscle fiber atrophy, mostly with predominance of type IIa fibers and absence or reduction of type IIb fibers with or without atrophy of type I fibers. Some patients showed occasional degeneration or necrosis of muscle fibers, central nucleus, opaque muscle fibers, and decreased enzyme activity. There was no difference in muscle pathology between MC and PMC, and both lacked specific changes compared to DMs, which have pyknotic nuclear clumps and nuclear accumulation, and periodic paralyses, which have vacuoles and tubular aggregates in some patients. NDMs are diagnosed based on symptoms and EMG before categorization based on genetic analysis, but a muscle biopsy is not necessary for diagnosis and should be performed only for research purposes.

### Genetic Analysis

NDMs are mainly related to the reduced activity of the CLC-1 channels or impaired inactivation of the Na_V_1.4 channels caused by *CLCN1* or *SCN4A* variants, which lead to sarcolemmal hyperexcitability manifesting as myotonia (34). The voltage-gated muscle CLC-1 channels mainly stabilize the resting membrane potential and inhibit the excitability of the sarcolemma. The ClC-1 channel is a dimer of two homologous subunits, each with its own ion-conducting pore, forming a unique double-barreled molecular structure. Each subunit consists of 18 transmembrane helices (A-R) and two tandem cystathionine-β-synthase (CBS1, 2) domains (28). Dominant *CLCN1* variants in DMC patients have a dominant negative effect (the negative impact of the mutated subunit on the co-expressed wild-type subunit) that causes a large shift in gating potential and prevents the ClC-1 channels from opening when required in repolarization, increasing the excitability of the membrane, manifested as myotonia (12). Recessive *CLCN1* variants in RMC patients lead to loss of function of the mutated subunit, which causes the accumulation of potassium ions and after depolarization bursts, manifesting as myotonia (35). In our study, 17 patients with MC included 10 with dominant *CLCN1* heterozygous variants and seven with recessive *CLCN1* compound heterozygous variants. A total of 19 different variants in the *CLCN1* gene were identified, mainly distributed on exons 8, 3, 12, among which exon 8 was located in the hot-spot region (17, 21). Combining these data with the patients' clinical symptoms and family history, the novel c.795T>G (p.D265E) missense variant is considered likely pathogenic.

The activation of Na_V_1.4 channels generates action potential (AP), and the fast inactivation after AP can prevent repetitive discharge, which ensures the physiological excitability changes of sarcolemma and normal skeletal muscle contraction. *SCN4A* encodes the pore-forming α-subunit of the Na_V_1.4 channel, which consists of four domains (DI-DIV), each with six transmembrane segments (S1–S6) (36). In our study, the L689F variant in DII S4–S5 and the V1293I variant in DIII S6 in three PMC patients could lead to impaired fast or slow inactivation of the Na_V_1.4 channel, persistent increased sodium current, and sustained sarcolemmal depolarization, causing increased excitability of the muscle fiber membrane and myotonia (1, 18, 19).

### Treatment

Sodium channel blockers (mexiletine, carbamazepine) are recommended to treat myotonia in NDM patients, which can slow down the depolarization speed of AP, thereby preventing repetitive Aps (37, 38). In this study, patients were treated with mexiletine or/and carbamazepine, and myotonia symptoms were partially improved. However, anti-myotonic drugs can unmask arrhythmias or even have pro-arrhythmic effects. ECG or Holter monitoring should be performed on patients during or before the use of drugs.

In conclusion, NDMs are a group of hereditary skeletal muscle ion channelopathies characterized by myotonia and caused by *CLCN1* or *SCN4A* variants, mainly including MC and PMC. MC and PMC have considerable phenotypic overlap. Facial stiffness and eye closure myotonia are more common in PMC, which may be accompanied by permanent weakness. Myotonic runs are found in EMG, and the muscle pathology of NDM lacks specific changes. Muscle biopsy is not necessary for diagnosis and should be performed only for research purposes. Genetic investigation contributes to identifying the subtype of NDM. Sodium channel blockers (mexiletine, carbamazepine) partially relieve the symptoms of myotonia.

## Data Availability Statement

The datasets presented in this study can be found in online repositories. The names of the repository/repositories and accession number(s) can be found below: ClinVar, VCV000623937.9.

## Ethics Statement

The studies involving human participants were reviewed and approved by the Ethics Committee of the Third Hospital of Hebei Medical University and were performed in accordance with the Declaration of Helsinki. Written informed consent to participate in this study was provided by the participants' legal guardian/next of kin. Written informed consent was obtained from the individual(s), and minor(s)' legal guardian/next of kin, for the publication of any potentially identifiable images or data included in this article.

## Author Contributions

QW collected literature and drafted the manuscript. JH made substantial contributions to manuscript revision. JH and QW designed the study and were responsible for data acquisition and analysis. ZZ and HS prepared muscle pathological figures. QB and NL prepared electromyography data. All authors reviewed the manuscript, agreed to be accountable for all aspects of the work, read and approved the final manuscript.

## Funding

This work was supported by the Doctoral Scientific Research Foundation of Qilu Hospital of Shandong University (QDKY2021BS02).

## Conflict of Interest

The authors declare that the research was conducted in the absence of any commercial or financial relationships that could be construed as a potential conflict of interest.

## Publisher's Note

All claims expressed in this article are solely those of the authors and do not necessarily represent those of their affiliated organizations, or those of the publisher, the editors and the reviewers. Any product that may be evaluated in this article, or claim that may be made by its manufacturer, is not guaranteed or endorsed by the publisher.
