# Supplementary material for: The Clinical, Myopathological, and Genetic Analysis of 20 Patients With Non-dystrophic Myotonia
Source: Front Neurol. 2022 Mar 8;13:830707. doi: 10.3389/fneur.2022.830707 (PMC8957821; doi:10.3389/fneur.2022.830707)
Supplement: Supplementary file 1 [file Data_Sheet_1.pdf]

Table 1s. Cardiologic data of 11 patients with non-dystrophic myotonia.

Pt. ID	Phenotype	Onset/ First-diagnosis/ Found cardiac involvement age (y)	Familial history of cardiac problems	Symptoms	ECG/Holter	Echocardiography	Medications used when cardiology investigations were performed
1	DMC	9/15/15	The father died of sudden cardiac death	–	Sinus tachycardia	Minor tricuspid regurgitation	–
2	DMC	19/21/21	–	Palpitations, chest tightness	Atrial premature beats	–	–
3	DMC	3/4/4	–	–	Sinus tachycardia	–	–
4	DMC	53.5/54/54	–	–	Ventricular premature beats	–	–
5	DMC	9/12/18	The mother complained of palpitations from the age of 26	Palpitations	Atrial premature beats	Minor mitral and tricuspid regurgitation	Mexiletine (100mg, 3×/day) and carbamazepine (100mg, 2×/day)
11	RMC	12/14/18	–	Palpitations, chest pain	Atrial premature beats, ventricular premature beats	–	Mexiletine (100mg, 3×/day)
12	RMC	9/12/12	–	–	Sinus arrhythmia	–	–
13	RMC	2/4/4	–	–	Sinus arrhythmia	–	–
14	RMC	1/14/18	–	Palpitations	Elevated heart rhythm variability	–	Mexiletine (100mg, 3×/day) and carbamazepine (200mg, 2×/day)
18	PMC	2/4/13	–	Chest tightness	Atrial premature beats, sinus tachycardia	–	Mexiletine (50mg, 3×/day) and carbamazepine (100mg, 2×/day)
19	PMC	5/20/20	–	Chest tightness, chest pain	Complete right bundle branch block	Minor tricuspid regurgitation	–

–, Negative or normal; y, year(s); ECG, electrocardiogram; Holter, 24-h Holter; DMC, autosomal dominant Thomsen's myotonia congenita; RMC, autosomal recessive Becker's myotonia congenita; PMC, paramyotonia congenita.

Table 2s. Muscle biopsy data of 17 patients with non-dystrophic myotonia.

Pt. ID	Phenotype	Age at biopsy(y)	Fiber diameter (μm)	Degeneration or necrosis muscle fibers	Central nucleus	Opaque muscle fibers	Enzyme activity	Selective muscle fiber atrophy or abnormal muscle fiber type distribution
1	DMC	15	30−100	occasional degeneration muscle fibers	–	–	–	atrophy of type I fibers, predominance of type IIfibers
2	DMC	21	60−120	–	–	–	focally decreased	two-type fiber grouping
3	DMC	4	20−40	degeneration muscle fibers	–	+	–	predominance of type I fibers
4	DMC	54	30−100	–	–	–	focally decreased	atrophy of type II fibers
5	DMC	12	50−120	–	–	–	–	predominance of type IIa fibers and absence of type IIb fibers
7	DMC	5	–	–	–	–	–	-
8	DMC	20	–	–	–	–	–	-
9	DMC	14	20−70	–	–	–	focally decreased	predominance of type IIa fibers and absence of type IIb fibers
10	DMC	27	20−120	occasional degeneration muscle fibers	–	–	focally decreased	predominance of type IIa fibers and absence of type IIb fibers
11	RMC	14	–	–	–	–	–	-
13	RMC	4	20−50	–	–	+	–	atrophy of type I fibers
14	RMC	14	10−90	occasional degeneration muscle fibers	–	–	–	predominance of type IIa fibers and absence of type IIb fibers
15	RMC	8	30−80	–	–	–	focally decreased	atrophy of type I fibers
17	RMC	20	40−110	–	+	–	–	atrophy of type I fibers, predominance of type II fibers
18	PMC	4	10−70	–	–	–	–	atrophy of type I fibers, predominance of type IIa fibers and absence of type IIb fibers
19	PMC	20	10−120	occasional degeneration and necrosis muscle fibers	++	–	–	predominance of type II fibers
20	PMC	5	10−80	–	–	–	–	predominance of type I fibers, atrophy of type IIa fibers and reduction of type IIb fibers

−, Negative or normal; +, positive; y, year(s); DMC, autosomal dominant Thomsen's myotonia congenita; RMC, autosomal recessive Becker's myotonia congenita; PMC, paramyotonia congenita.

Table 3s. Pathogenicity analysis of mutant genes.

Gene	Nucleotide change	Protein change	Inheritance mode	Exon	Structural position	Pathogenicity analysis
CLCN1	c.T920C	p.F307S	AD	8	I	PS1+PM1+PM2+PP1+PP3+PP4=P
CLCN1	c.2527C>T	p.L843F	AD	22	CBS2	PS1+PP1+PP3+PP4=LP
CLCN1	c.892G>A	p.A298T	AD/AR	8	H-I	PS1+PM1+PM2+PP3+PP4=P
CLCN1	c.350A>G	p.D117G	AD	3	B	PS1+PS2+PM2+PP3+PP4=P
CLCN1	c.1261dupC	p.R421fs	AD	12	-	PVS1+PS1=P
CLCN1	c.1679T>C	p.M560T	AD	15	Q	PS1+PS2+PM2+PP3+PP4=P
CLCN1	c.214_215delAG	p.R72fs	AD	2	-	PVS1+PS1=P
CLCN1	c.2362C>T	p.Q788X	AD	19	CBS1- CBS2	PS1+PP1+PP4=LP
CLCN1	c.2207C>T	p.T736I	AR	18	CBS1- CBS2	PS1+PM3+PP4=LP
CLCN1	c.433G>T	p.A145S	AR	3	B-C	PS1+PM2+ PM3+PP3+PP4=P
CLCN1	c.1277C>A	p.T426N	AR	12	L-M	PS1+PM2+PM3+PP3+PP4=P
CLCN1	c.762C>G	p.C254W	AR	6	F-G	PS1+PM2+PP3+PP4=LP
CLCN1	c.962T>A	p.V321E	AR	8	I-J	PM2+PM3+PP3+PP4=LP
CLCN1	**c.795T>G**	**p.D265E**	AR	7	G	PM2+PM3+PP3+PP4=LP
CLCN1	c.1872G>T	p.E624D	AR	16	CBS1	PS1+PM2+PP3+PP4=LP
CLCN1	c.857T>A	p.V286E	AR	8	H	PS1+PM3+PP3+PP4=LP
CLCN1	c.1012C>T	p.R338*	AR	9	-	PVS1+PS1=P
CLCN1	c.1389ins T	p.F463fs	AR	12	-	PVS1+PS1=P
CLCN1	c.2330del G	p.G777fs	AR	19	-	PVS1+PS1=P
SCN4A	c.3877G>A	p.V1293I	AD	22	DIII S6	PS1+ PS2+ PM2+ PP3+PP4=P
SCN4A	c.2065C>T	p.L689F	AD	13	DII S4-S5	PS1+ PS2+ PM2+ PP3+PP4=P

ClC-1, NM_000083; *SCN4A*, NM_000334; Bold text, novel mutation; AD, autosomal dominant; AR, autosomal recessive; P, pathogenic; LP, likely pathogenic. The pathogenicity of gene variants was judged by the American College of Medical Genetics and Genomics (ACMG) Standards [Richards et al., 2015].
